# The Headphone and Loudspeaker Test – Part I: Suggestions for controlling characteristics of playback devices in internet experiments

**DOI:** 10.3758/s13428-022-01859-8

**Published:** 2022-05-17

**Authors:** Yves Wycisk, Reinhard Kopiez, Jakob Bergner, Kilian Sander, Stephan Preihs, Jürgen Peissig, Friedrich Platz

**Affiliations:** 1 Hanover University of Music, Drama and Media, Neues Haus 1, 30175 Hannover, Germany; 2 Institute of Communications Technology, Leibniz University Hannover, Hannover, Germany; 3 Department of Musicology, Music Education and Aesthetics, University of Music and Performing Arts, Stuttgart, Stuttgart, Germany

**Keywords:** Playback characteristics, Internet experiment, Remote testing, Confounding variables, Control variables, Level adjustment, Mono/stereo playback

## Abstract

In internet experiments on auditory perception, playback devices may be a confounding variable reducing internal validity. A procedure to remotely test multiple characteristics of playback devices does not currently exist. Thus, the main goals of this study were to (i) develop and (ii) evaluate a comprehensive, efficient, and easy-to-handle test procedure for the reliable control and identification of playback device characteristics in online experiments. Based on a counting task paradigm, the first part of the Headphone and Loudspeaker Test (HALT–Part I) was developed with which researchers can standardize sound level adjustments, detect stereo/mono playback, and assess lower frequency limits. In a laboratory study (*N* = 40), HALT–Part I was evaluated with four playback devices (circumaural and intra-aural headphones; external and laptop loudspeakers). Beforehand, the acoustical properties of all playback devices had been measured (e.g., sound pressure level, frequency response, total harmonic distortion). The analysis suggested that HALT–Part I has high test–retest reliability (*r*_tt_ = .90 for level adjustment and *r*_tt_ = .79 for stereo/mono detection) and is an efficient (3.5 minutes for completion) method to remotely test playback devices and listening conditions (sound level, stereo/mono playback). The procedure can help improve data quality in internet experiments.

**Supplementary Information:**

The online version contains supplementary material available at 10.3758/s13428-022-01859-8.

## Introduction

Computer-based experiments have revolutionized data collection in research since the 1970s. Compared to paper-and-pencil methods, computer-based studies show clear advantages: direct data entry, measurement of response time, and interactivity. Additionally, socially desirable response behavior can be avoided when the experimenter is not physically present. A major advantage of the new technology is that stimuli can be presented in a standardized, randomized, and controlled manner (Musch & Reips, 2000). Additionally, the use of the Hypertext Transfer Protocol (HTTP) via the World Wide Web (WWW), which was developed in the early 1990s by the European Organization for Nuclear Research (CERN; Musch & Reips, 2000), opened up a new and promising possibility for psychological experiments beyond the laboratory situation (Birnbaum, 2004). The WWW made it possible to carry out psychological surveys and experiments without geographical constraints. This development could be regarded as a revolutionary advance in psychological research (Musch & Reips, 2000). To the best of our knowledge, the first internet-based experiment published in a scientific journal was carried out by Krantz, Ballard, and Sher in 1995 on the topic of predictors of female attractiveness (Krantz et al., 1997). Even in this early phase, the researchers recognized the risk of possible confounding variables that would be difficult to control due to the data collection method. For that reason, the authors compared their WWW-collected data with data from the laboratory (Krantz et al., 1997).

In psychological experiments on auditory perception, the stimuli that participants hear are of central importance. A mandatory precondition for investigating stimulus-dependent response behavior is to keep the playback situation unchanged between participants.

Ideally, all participants should receive the stimuli under identical acoustical conditions. If this precondition is not feasible, confounding variables such as different playback devices and characteristics should be controlled for or avoided (Kirk, 2003). However, in online experiments using auditory stimuli, the type of headphones or loudspeakers that participants use usually remains unknown. The sound-transducing equipment may influence the participants’ responses significantly. Additionally, test conditions vary due to different acoustic listening situations. For example, Kopiez et al. (2016) investigated whether participants could distinguish short musical examples performed by real-life orchestras from simulations of the same passage produced via orchestra sample libraries. Experts (among them, sound engineers and producers) performed better in the discrimination task than non-expert listeners. However, it was unclear whether the superior performance of experts was truly based only on better listening expertise. For instance, experts in the study might have used playback devices with superior characteristics compared to the average participant. Without knowledge of the playback devices, it remains difficult to interpret the findings unambiguously. This raises the question of how to control for the confounding variable of listening conditions. The way in which characteristics of playback devices can influence participants’ responses could provide an indication of possible test procedures for the headphone and loudspeaker test (HALT). Unfortunately, to the best of our knowledge, there are only a few studies on this topic. Previous research dealing with the characteristics of sound transducers mainly focused on the subjects’ judgments regarding reproduction quality (Chon & Sung, 2010; Leong et al., 1999; Letowski, 1989; Olive et al., 2013; Toole, 1982). In one study (Stupacher et al., 2016), the relationship between audio features, perceived groove, and sensorimotor synchronization was examined. The variability of energy in frequency bands below 100 Hz was found to be the best predictor. Burger et al. (2013) reported that spectral flux in the range of 50–100 Hz was correlated positively with the speed of head movement. Whether certain stimulus properties are heard or can be transduced depends on the capabilities of the playback device used. Together, the stimulus and the playback device form an inseparable unit. For example, when low-frequency components of a stimulus are important, the low-frequency capability of the playback device automatically becomes important as well. The true stimulus is generated only during playback. Playback devices should, therefore, be controlled.

In another example, Todd and Cody (2000) found evidence that sound pressure level (SPL) influenced the activation of the vestibular system, resulting in an urge to move (groove sensation). There may be a minimum volume level for rock and dance music at which these musical genres produce their characteristic effects. Todd and Cody (2000) identified a sound level threshold of circa 90 dBSPL (A) as the preferred level for the full experience of rock and dance music. Despite all of the abovementioned indicators concerning the influence of playback devices, there is currently no objective, comprehensive, and efficient method for the remote determination of multiple characteristics of listening devices. However, there are studies that deal with the identification of headphones and loudspeakers (Milne et al., 2020; Woods et al., 2017). Moreover, Pankovski (2021) developed a method to verify dichotic playback. Controlled online studies have become even more important in light of the COVID-19 pandemic, as researchers relocate experiments from the laboratory to the internet. Finally, the need for standards and control mechanisms for perceptual internet experiments is also reflected in initiatives such as the ASA P&P Task Force on Remote Testing (www.spatialhearing.org/remotetesting). Additionally, Eerola et al. (2021) highlight the importance of controlling the playback conditions and give a comprehensive overview of online data collection in auditory contexts.

### Study aims

1. Our main research aim was the development of a reliable and efficient headphone and loudspeaker test to remotely test playback device characteristics and playback conditions such as sound level and stereo/mono playback.
2. To reduce dropout rates, we aimed for the test to be as short as possible. We decided that the entire HALT procedure should be accomplished in less than 10 minutes. According to Reips (1997), the dropout rate can be reduced if participants are given a feeling of commitment to participate. This approach is called the high-hurdle technique (Reips, 2012). The HALT procedure can be seen as a high-hurdle technique, but it is unclear how the dropout rate would be influenced.
3. Another aim was the development of objective tasks to provide a statistically criteria-related evaluation. Decisions regarding the exclusion and inclusion of participants based on self-reports were to be avoided.
4. We also aimed to develop a test procedure that covered multiple characteristics of playback devices with a variety of different listening tasks.
5. The designed test procedure was to be validated in a controlled laboratory situation and acoustical features of playback devices documented.

Our aim was not to develop an overall quality index for playback devices by using certain features to calculate summary scores. Depending on the content of the research questions, the prerequisites for studies can be very different. We sought to test or control individual characteristics (e.g., standardize sound level adjustments, detect mono/stereo playback and interchanged channels, and assess the lower-frequency limit) to address the individual needs of researchers in constructing their studies. In our opinion, a quality index would not address those individual needs. Nonetheless, the method proposed does not provide a full characterization of the playback devices. Consequently, it is not meant to be used for audiometry testing.

In “The Headphone and Loudspeaker Test – Part II” (Wycisk et al., 2021) we address screening methods to detect headphone and loudspeaker playback based on perceptual tasks and statistical procedures.

## Method

### Experimental setup and procedure

It is likely that the average person participating in an online study neither sits in an acoustically optimized room nor uses high-end loudspeakers or headphones. Thus, it was decided that HALT should perform in ordinary non-optimized listening environments and with sound devices of diverse characteristics. For that reason, the laboratory experiment took place in a non-optimized laboratory room of the Hanover Music Lab (HML; see S1 in the Supplemental Materials for room acoustical measurements) with a variety of low- to average- and high-quality transducers. In general, we believe that it is difficult to assign a quality level to a device, as this involves weighting of playback device characteristics. Depending on the purpose, certain characteristics can be of differential importance. The assigned quality level in this study is only a subjective classification. A precise assignment is negligible, since we do not want to develop a quality index for playback devices. Due to the need for the length of the test procedure, only four devices were used:

- Beyerdynamic DT 770 Pro 250 Ohm, closed circumaural, high-quality headphones.
- No-name earbuds, open, intra-aural, low-quality headphones.
- A pair of Yamaha HS8M loudspeakers (near field monitor) of average quality.
- Apple MacBook Pro, 13” (Retina, early 2015) low-quality loudspeakers/laptop.

The opening angle of the laptop was 110°. The measurement device used was a head and torso simulator (HATS, GRAS 45BC-11 KEMAR). The loudspeakers and the HATS created an isosceles triangle with a long edge length of 1.11 m. All devices and furniture positions were marked with colored tape on the carpet floor to guarantee reliable reconstruction of the setup (see S2 in the Supplemental Materials).

Data collection in the laboratory was based on the browser-based survey platform SoSci Survey (www.soscisurvey.de; Leiner, 2020). A complete retest using all four devices was conducted. After giving demographic information, participants started with the average-quality loudspeaker condition (Yamaha HS8M), followed by the low-quality loudspeaker/laptop (Apple MacBook Pro), high-quality headphones (Beyerdynamic DT 770 Pro), and low-quality no-name headphones (see S3 in the Supplemental Materials for the procedure). During the experiment, the experimenter and the participant were located in separate rooms. Digital levels were monitored and recorded using a second screen in the experimenter’s room (split screen extension of the participant's computer). The digital amplification values for the loudspeaker and headphone playback were provided by the RME Totalmix FX software (version 1.65; Audio AG, 2020). In the laptop condition, Apple’s Audio MIDI Setup application was used to display the playback amplification. Each listening session lasted approximately 90 minutes, including instructions, pauses, and retests.

### Stimuli and task development

We developed stimuli and associated tasks to control the basic level adjustment (A.1), to check for level invariances and unwanted level manipulations (A.2), to check for mono and stereo (A.3), and to estimate the lower frequency limits of playback devices (A.4). As the main principle for stimulus construction, a counting paradigm was used to set up a comprehensive test procedure. All stimuli were created on an Apple MacBook Pro*,* 13″ (mid-2012) using Logic Pro X. In general, researcher-developed stimuli were limited to −1 dBFS (decibels relative to full scale, true peak) to avoid clipping through the Gibbs phenomenon (Oppenheim & Schafer, 2014). For each condition and counting task, a separate stimulus was created to avoid the influence of memory effects on responses. To prevent forward and backward masking, a gap of around 200 ms between auditory events within stimuli was included (Plack, 2010). Most of the stimuli use noise as a main component. Pink noise (20 Hz to 20,000 Hz) was used, as its power spectral density is similar to music. Additionally, the signal covered a wide frequency range. As a result, a wide transmission range of the playback devices and also local peaks was made audible. Responses were collected via the SoSci Survey (www.soscisurvey.de) browser interface (Leiner, 2020). To respond to the counting tasks, subjects had to enter numerical values on the website. All tasks with the associated stimuli can be tested in a demo version of the HALT (http://testing.musikpsychologie.de/HALT_demo_no_screening/). The program code (R package) is freely available on GitHub (https://github.com/KilianSander/HALT).

#### (A.1.) Item development for basic level adjustment

Three stimulus classes/types (M = music, N = noise, L = loop) were used to develop test items (stimuli and task) for adjusting the volume. Stimulus M was an excerpt of 30 s from the song “Menschen Leben Tanzen Welt*”* (Jim Pandzko, 2017). This song is quite characteristic of pop music production, including low-frequency enhancement and strong amplitude compression (long-term LUFS [loudness units relative to full scale] = −8.4, range LU [loudness units] = 6, Level = −0.2 dBFS true peak). The task was to listen to the excerpt and set the volume to a personally comfortable level, which the participant would prefer in an online study.

The second stimulus (N) consisted of 12 low-level pink-noise segments at −46 dBFS true peak. The participants were instructed to adjust the volume in such a way that the noise segments could be barely heard but were still perceivable. This stimulus was used to set the baseline for the subsequent loop method (explained in the next section). The general idea of this task was that the participants would set the level just above the background noise in the room. As we are not aware of any studies on the topic, the level of −46 dBFS true peak was chosen arbitrarily. We were aiming for a final playback level of around 85 dBSPL (sound pressure level, A-weighted) including the 1 dB gain reduction to avoid clipping through the Gibbs phenomenon. The A-weighting accounts for the human perception, while Z-weighting represents a flat frequency response.

Stimulus L was comprised of low-level and high-level pink noise segments. Low-level noise segments were presented at irregular time intervals and always had the same level of −46 dBFS true peak. High-level segments were regularly presented at a level of −1 dBFS true peak to keep participants from increasing the volume. A loop stimulus always contained a true/correct number of noise segments (low-level and high-level). The task was to count all the heard segments. In this way, we created an objective decision criterion (true number/correct number of segments reported, too many/too few reported) to assume correct and incorrect sound level adjustments. When participants tried to solve the listening task by increasing the volume, the unpleasant loud noise events had a deterring function. If participants reported too many events, they had to repeat the task and were prompted to listen more carefully. If a participant reported too few counts, it was assumed that the volume was set too low, which meant that the task could not be solved correctly. Accordingly, participants were prompted to increase the volume by the smallest possible value and to repeat the task. If the true number of noise segments were reported, the participant progressed to the next task. Through the direct response in form of prompts, a feedback loop was created that allowed control of sound level adjustments.

After each of the three types of stimuli (M, N, L), participants were asked to rate the perceived loudness of a pop song (Jim Pandzko, 2017) on a three-point rating scale (too soft, comfortable, too loud). In addition, in all four playback conditions, the digital amplification values set by the participants were documented. In a later stage, the adjusted sound levels in all three adjustment-method conditions can be compared.

#### (A.2.) Item development for determining participants’ adjustment accuracy/manipulation check

The loop method (consisting of the loop stimulus and the loop task) described in the previous section guaranteed only a minimum volume. However, after successful completion of the loop method (true number of noise segments was reported), it was still unknown how loud or how accurate the volume was adjusted above the minimum volume. To build a method to assess the adjustment accuracy in internet experiments, we used a stimulus comprised of pink noise events at different levels (−52/−46/−40 dBFS true peak). Participants had to count all noise events they perceived. Since the participants previously went through the loop method (that ensured audibility of noise segments at −46 dBFS true peak), we assumed that all participants would hear the noise events at −46 dBFS true peak and louder (−40 dBFS true peak).

As all events in the stimulus were present in a different quantity, conclusions could be drawn from the response behavior as to which levels could not be heard by the participants.

The following series of events serves as an example: 3 × −52 dBFS true peak, 4 × −46 dBFS true peak, and 2 × −40 dBFS true peak (nine noise events in total). There are two ways to use the information obtained from this task. One is to check the accuracy of the set volume. Therefore, the task has to be presented directly after completing the loop method for sound level adjustment. If the participants identify nine events, every noise segment can probably be heard, meaning the volume is set too loud. If six events are counted, the −52 dBFS segments probably cannot be heard, resulting in the setting being called “accurate.” If only two events are counted, the volume presumably is set too low, although the loop method was completed. The participants may have correctly solved the loop task by chance. We apply moderate criteria (± 1 counts), classifying everyone in our example who counts five, six, or seven events as accurate. Participants who count more than seven are classified as “too loud” and those who count less than five as “too soft.” As the number of noise events in each condition and for each level is different, the classification criteria are applied to different thresholds in each condition.

Another way of using the counting responses is to detect possible unwanted level manipulations. The task is to be presented repeatedly at a later time. The first accuracy measurement serves as a baseline to help determine if the level settings have been manipulated. The second measurement is then used to identify whether the test taker is classified in the same group again (too soft, accurate, or too loud). If so, it can be assumed that the volume remained unchanged. In case of a volume change, the direction of a possible group-change indicates whether the volume was reduced (from “too loud” to “accurate,” from “accurate” to “too soft,” or from “too loud” to “too soft”) or increased (from “too soft” to “accurate,” from “accurate” to “too loud,” or from “too soft” to “too loud”). However, in our laboratory study, participants were instructed not to change the volume during the survey. The experimenter regularly checked for compliance. To check whether HALT could detect volume changes, we simulated two volume manipulations. We refer to the original stimulus set as condition 0 dB. In Duplicate A of the set, the overall level of the stimulus set was increased by 3 dB (+3 dB condition). In Duplicate B, the level was decreased by 3 dB (−3 dB condition). For all playback device conditions and level manipulations (−3 dB, 0 dB, +3 dB), there was one trial each.

#### (A.3.) Item development to check for mono/stereo playback settings

The stimulus consisted of pink noise events (−1 dBFS true peak) that alternated irregularly between the left and right channel. The noise never sounded on both channels at the same time. Between the two stereo channels, the number of events always differed for every playback device condition to avoid memory effects. The task was to count all audible noise segments on the right channel only. There was one trial for each playback device condition. In the case of mono playback, all noise events would have been audible on the right channel. As a result, a participant would have reported the total number of all noise segments. In the presence of interchanged channels or difficulties with right-left discrimination, we expected the participant to report the number of noise events from the left channel. If the number entered was equal to the number of noise events on the left channel, it was assumed that the channels were swapped. To control for difficulties with right-left discrimination, we created a visual task in which participants had to indicate the position of a circle relative to a triangle.

#### (A.4.) Item development to estimate the lower-frequency limits of playback devices

The stimuli consisted of randomly presented pure tones (−1 dBFS true peak) located between regularly presented sections of loud pink noise (−1 dBFS true peak). Again, the loud noise was added to prevent the subjects from increasing the volume to solve the task. The task gives an estimate of what the sound transducer can reproduce in a best-case scenario, when the capabilities of the playback devices are pushed to the limit. We, therefore, chose a high level for the pure tones (−1 dBFS true peak). In order to keep the workload low, we selected four frequencies (20, 60, 100, and 140 Hz) and tested their audibility in subtasks. Participants were asked to indicate the total number of pure tone events that they had heard. There was only one trial for each frequency. We assumed that the pure tones could only be heard if the playback device was capable of reproducing the respective frequency adequately. For interpretation, the entire reproduction chain and the perception of the participants has to be taken into account. As a control procedure, the lower-frequency limits of every transducer determined by the HATS measurements (see next section) were compared with those determined by HALT.

### Electroacoustical analysis of playback devices used in the laboratory study

To assess the relationships between the results of the perceptual tasks and the electroacoustic properties of the reproduction setups, we measured total harmonic distortion (B.1), frequency responses and limits (B.2), linearity (B.3) and the stimulus level (B.4) for each playback device. We used a GRAS 45BC-11 KEMAR Head and Torso Simulator (HATS) with anthropometric pinnae and low-noise ear simulator in combination with an Audio Precision APx525 measurement system. The analysis and evaluation were conducted with a routine scripted with MATLAB. The electroacoustic parameters were selected according to the *International Electrotechnical Commission (IEC)* standard IEC 60268-5 (2003) for loudspeaker and the standard IEC 60268-7 (2010) for headphone measurement, and could be derived from logarithmic sweep measurements (Farina, 2000). To investigate the devices’ behavior under conditions comparable to the experimental conditions, we chose an open loop measurement approach (Begin, 2020) which could be interpreted as a sequential dual-channel fast Fourier transform (FFT) method (Müller & Massarani, 2001). Specifically, test signals were created as audio files, which were then transferred and played back with the actual reproduction setup (i.e., MacBook Pro ➔ RME Babyface ➔ loudspeaker/headphones; see S4 in the Supplemental Materials for details of the signal chain).

#### (B.1.) Analysis of total harmonic distortion

An analysis of harmonic distortion as a function of digital amplification gain (in dB) was carried out. Based on this method, the optimal voltage for driving the individual transducers with an acceptable influence of artifacts could be determined. In the case of loudspeaker reproduction, stereo presentation was assumed; for example, crosstalk from the right loudspeaker to the left ear was taken into account, and resulting total harmonic distortions (THD) were summed up accordingly. For better clarity, the THD is depicted in Table 1 as the average for left and right ears in % for specific frequencies.

**Table 1:** Total harmonic distortion in % for selected frequencies and amplification gain settings of the loudspeaker pair Yamaha HS8M

Gain/dB	dBSPL	THD/%							
	1 kHz	125 Hz	250 Hz	50 Hz	1 kHz	2 kHz	4 kHz	8 kHz	Mean
+3	91.7	1.7	2.6	3.3	7.7	1.0	0.3	1.0	2.5
0	92.4	1.3	2.5	3.3	6.8	0.9	0.3	0.9	2.3
−3	89.7	1.4	2.2	3.2	6.4	0.8	0.3	0.8	2.2
−6	86.8	1.4	2.2	3.1	6.0	0.9	0.4	1.0	2.1
−12	80.9	2.1	2.5	2.8	5.3	1.0	0.7	1.8	2.3
−20	75.6	6.4	5.8	3.4	3.9	2.0	1.7	4.1	3.9
−30	58.2	18.6	21.4	9.1	5.3	4.5	4.6	11.5	10.7
−40	53.3	61.5	44.9	40.4	14.7	14.8	16.9	37.2	32.9

Table 1 shows that the minimum mean THD of the Yamaha HS8M for the selected frequencies occurred for a digital playback level of −6 dBFS. For lower levels (−12...−40 dB) the THD values increase again, as the noise at multiples of the fundamental frequency is misinterpreted as harmonic distortion. This behavior is due to the THD calculation algorithm, which is based on short-time Fourier transform (Farina, 2000). The best-case digital gain settings concerning the resulting THD for all reproduction devices are shown in Table 2.

**Table 2:** Aggregated best-case THD in % for the reproduction devices depending on the digital gain settings and resulting SPL at 1 kHz

Transducer	Gain/dB	dBSPL	THD/%							
		1 kHz	125 Hz	250 Hz	500 Hz	1 kHz	2 kHz	4 kHz	8 kHz	Mean
Loudspeaker	−6	86.8	1.4	2.2	3.1	6.0	0.9	0.4	1.0	2.1
Laptop	−12	88.3	75.5	45.9	12.8	11.9	1.7	2.2	1.7	21.7
Headphones, high-quality	−12	90.6	0.3	0.1	0.1	0.1	0.1	0.2	0.3	0.1
Headphones, low-quality	−12	90.2	3.7	4.5	0.5	0.2	0.4	0.1	0.1	1.4

The loudspeaker gave higher THD values than both headphone types, as expected given the relatively small membrane surface and displacement (Klippel, 2006). The excessive mean THD for the laptop (MacBook Pro) occurred mainly for low to mid-frequencies (0.1–1 kHz). However, as shown in the analysis of the frequency response (Fig. 2), the sound pressure generated over this frequency range was very low.

#### (B.2.) Analysis of frequency response and limits

**Fig. 1:**
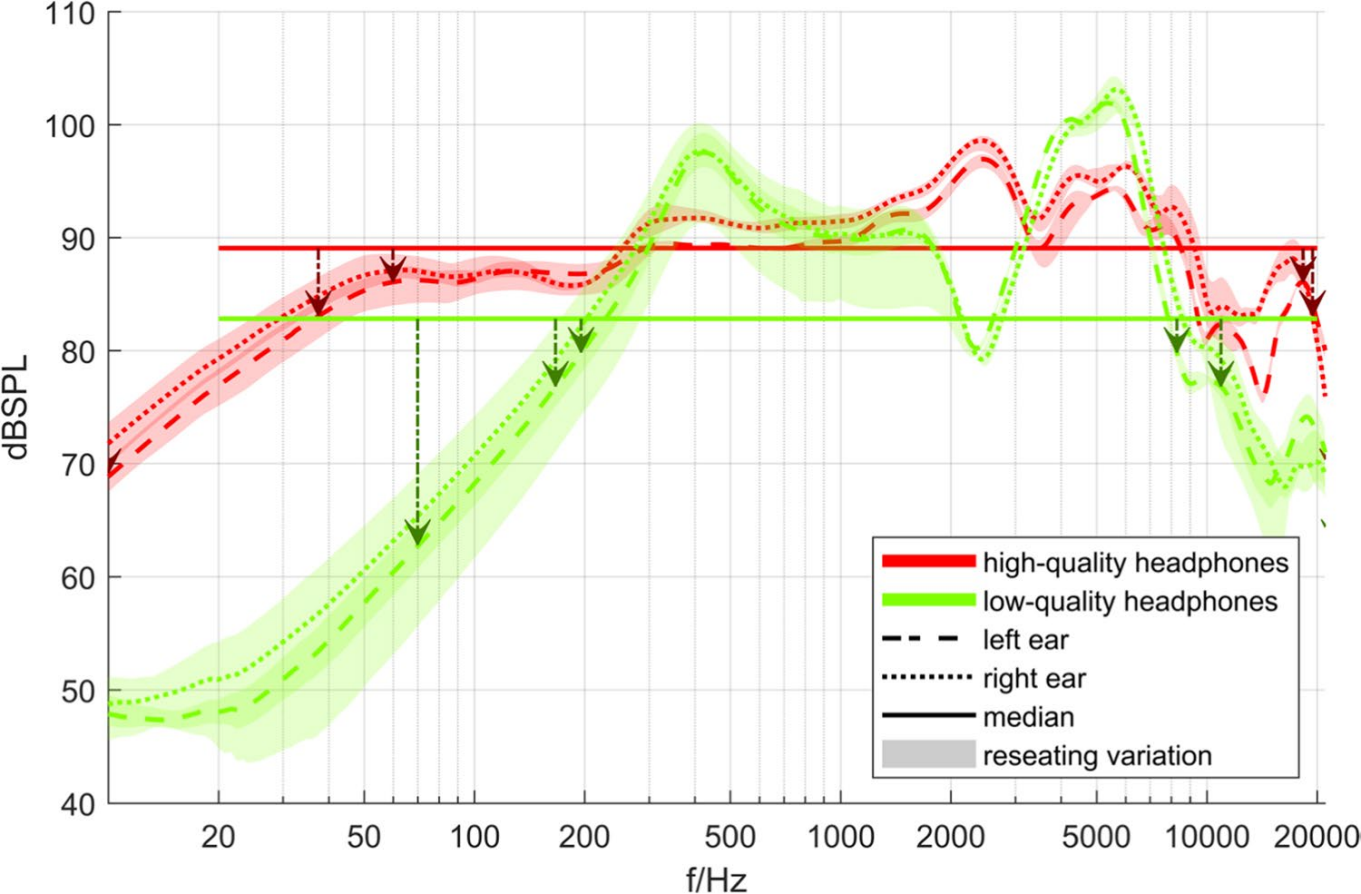
Magnitude spectra of the responses of the headphones with reseating variations, median levels, and frequency limits. *Note.* The vertical arrows indicate at which point the magnitude of the transfer function fell below the median by 3, 6, and 20 dB

**Fig. 2:**
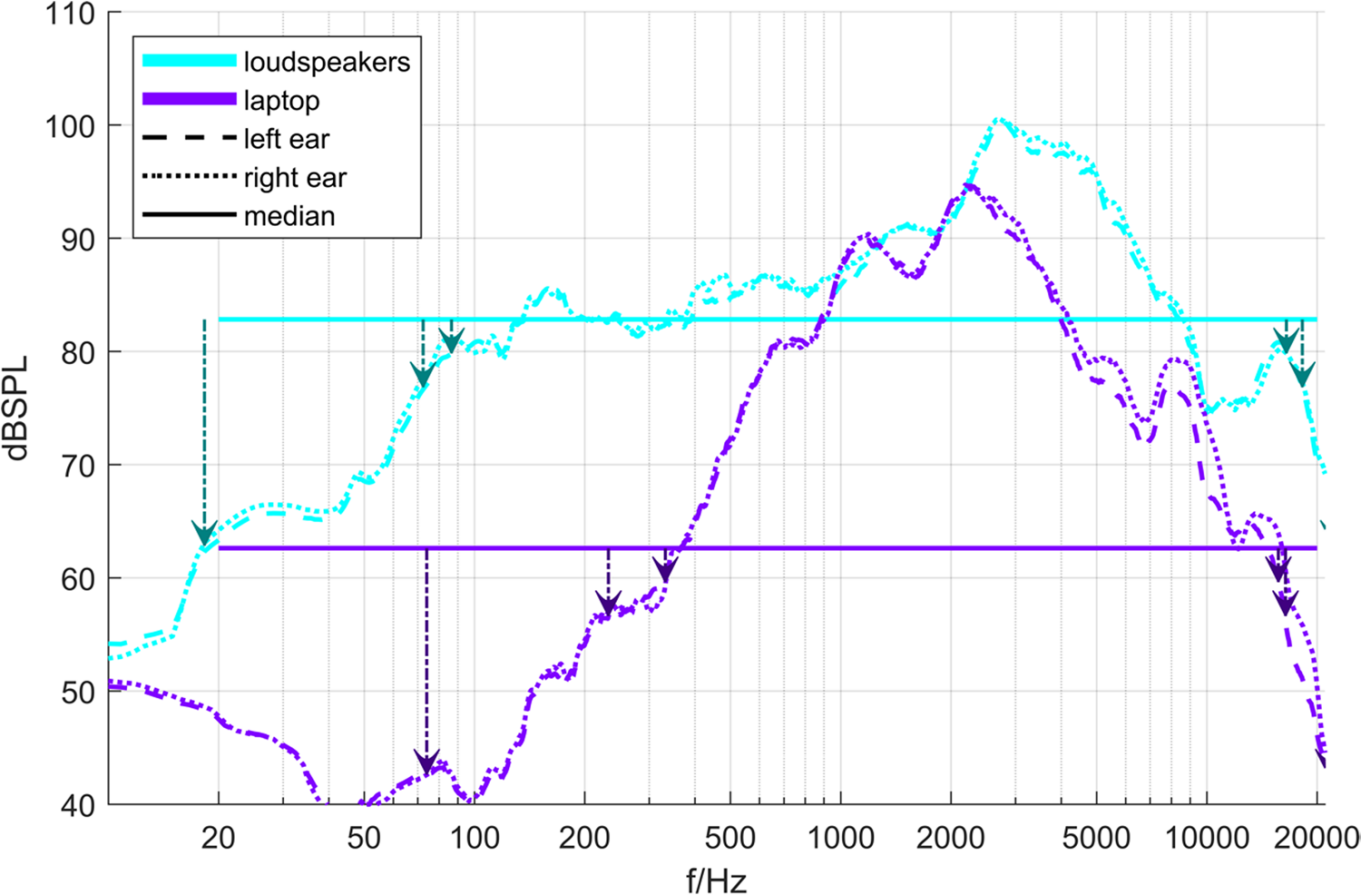
Magnitude spectra of the responses of the loudspeakers with median levels and frequency limits. *Note*. The vertical arrows indicate at which point the magnitude of the transfer function fell below the median by 3, 6, and 20 dB

The analysis of the frequency response in the following section is based on the magnitude spectra of the transfer functions of the individual devices. These can be found in Fig. 1 for the headphones and Fig. 2 for the loudspeakers. Both figures show the transfer functions for the left and right ears separately as third-octave smoothed magnitude responses. Figure 1 shows the range of five reseating measurements (taking off and putting on the headphones to account for positioning effects) of the headphones as shaded areas. The respective response curves denote the complex mean. The bold horizontal lines indicate the logarithmically sampled median magnitudes of the transfer functions in the range between 20 Hz and 20 kHz while frequency limits at which the magnitude fell below the median by 3, 6, and possibly even 20 dB are marked with vertical arrows. As stereo reproduction was used in all cases, the loudspeaker responses in Fig. 2 include crosstalk contributions (e.g., from the left speaker to right ear).

#### (B.3.) Analysis of linearity

**Fig. 3:**
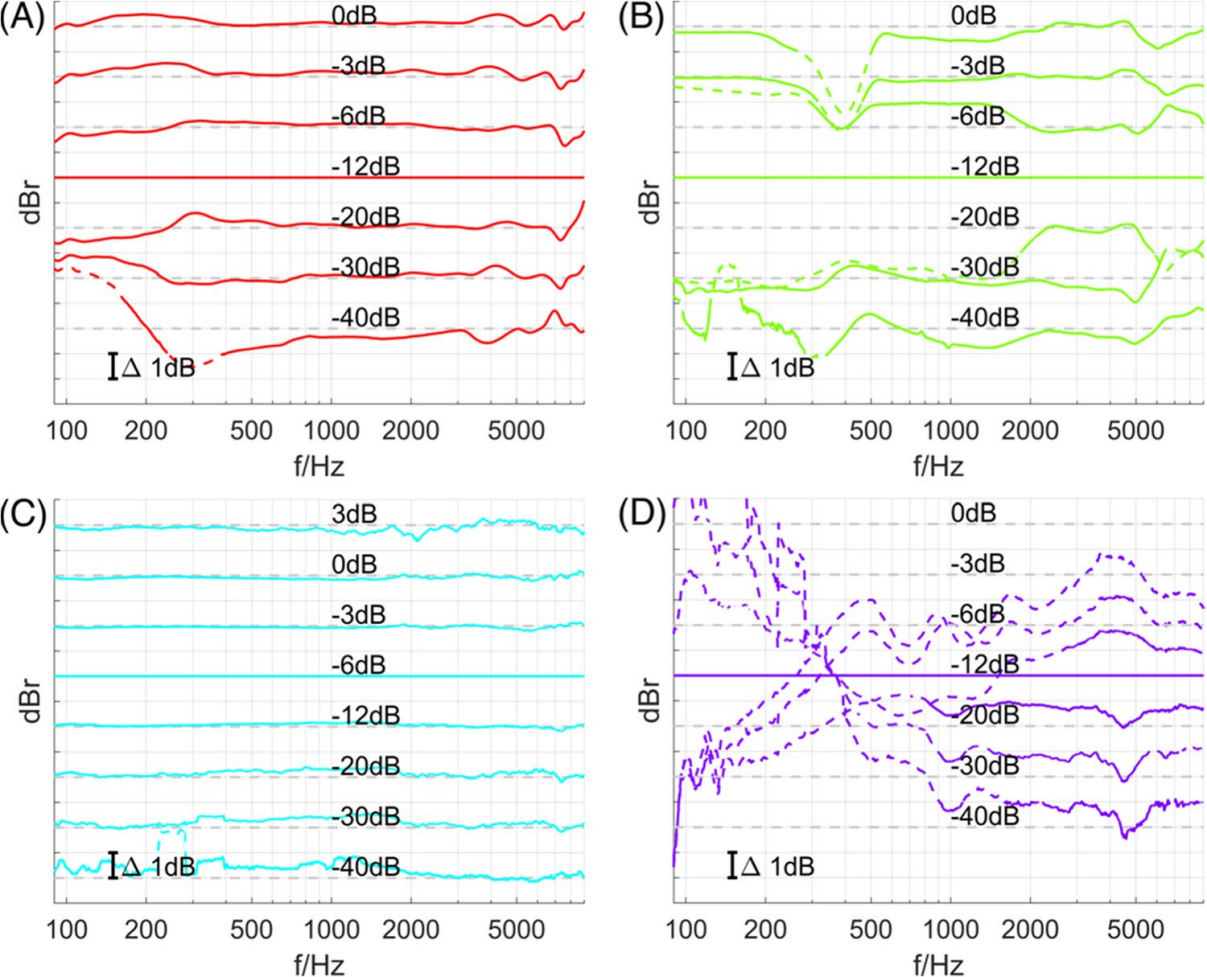
Analysis of deviations from reference response at various amplifications of the devices under test: high-quality headphones – Beyerdynamic DT 770 Pro (**A**), low-quality headphones – no-name earbuds (**B**), loudspeakers – Yamaha HS8M (**C**), laptop – MacBook Pro early 2015 (**D**)

The frequency responses were obtained for input voltages giving the best THD. However, investigations of other input voltages showed areas of nonlinear behavior of the respective devices, namely, areas in which changes in input voltage did not lead to the same changes in the acoustic output. This behavior can be explained by the loss of force when the voice coil leaves the magnet gap at high displacements (Klippel, 2006). Besides, it was expected that electronic consumer devices, such as the audio output of the laptop (MacBook Pro), contain integrated nonlinear dynamic processing such as compressors, expanders, and limiters to subjectively enhance the output of the low-quality built-in loudspeaker. Because there is no total control over the equipment used in online listening test scenarios, the influence of nonlinear behavior should be considered. Figure 3 shows the linearity of the devices under test.

The plots show the deviations of the magnitude responses for various gain settings relative to the response with the best THD. The curves are separated for better visibility. The dashed gray lines denote the respective 0 dB line for each gain. Perfect linearity would result in straight horizontal curves. Frequency areas with deviations beyond ±1 dB are marked with dashed curves. The average- to high-quality devices on the left side (panels **A** and **C**) showed only small deviations in magnitude response for most gain settings. It could be expected that the timbre of the reproduction would not vary with increasing or decreasing gain and level. In contrast, the low-quality devices (panels **B** and **D**) showed highly varying magnitude responses across different gain settings. In particular, the laptop (MacBook Pro, purple curves in the bottom right subfigure) showed large deviations throughout the investigated frequency and gain range. The measurements revealed that nonlinearities—in this case a mismatch between amplification gain and acoustic level—varied with both frequency and output voltage. This leads to the conclusion that the timbre of the reproduced audio stimuli might vary with gain setting or level. However, this observation is quantified according to physical acoustics while the perception of reproduced stimuli may lead to smaller and/or other deviations.

#### (B.4.) Analysis of stimulus levels

The previous electroacoustic analysis dealt with the individual reproduction systems independent of the specific stimuli. Subsequent investigations were related to the actual musical stimulus (stimulus M) that was used for the level adjustment process (see stimulus M in the section *Item development for basic level adjustment* [A.1]). Keeping the previously analyzed acoustical properties in mind—namely magnitude spectra, harmonic distortion, and linearity—it was possible to analyze the resulting overall sound pressure level.

**Fig. 4:**
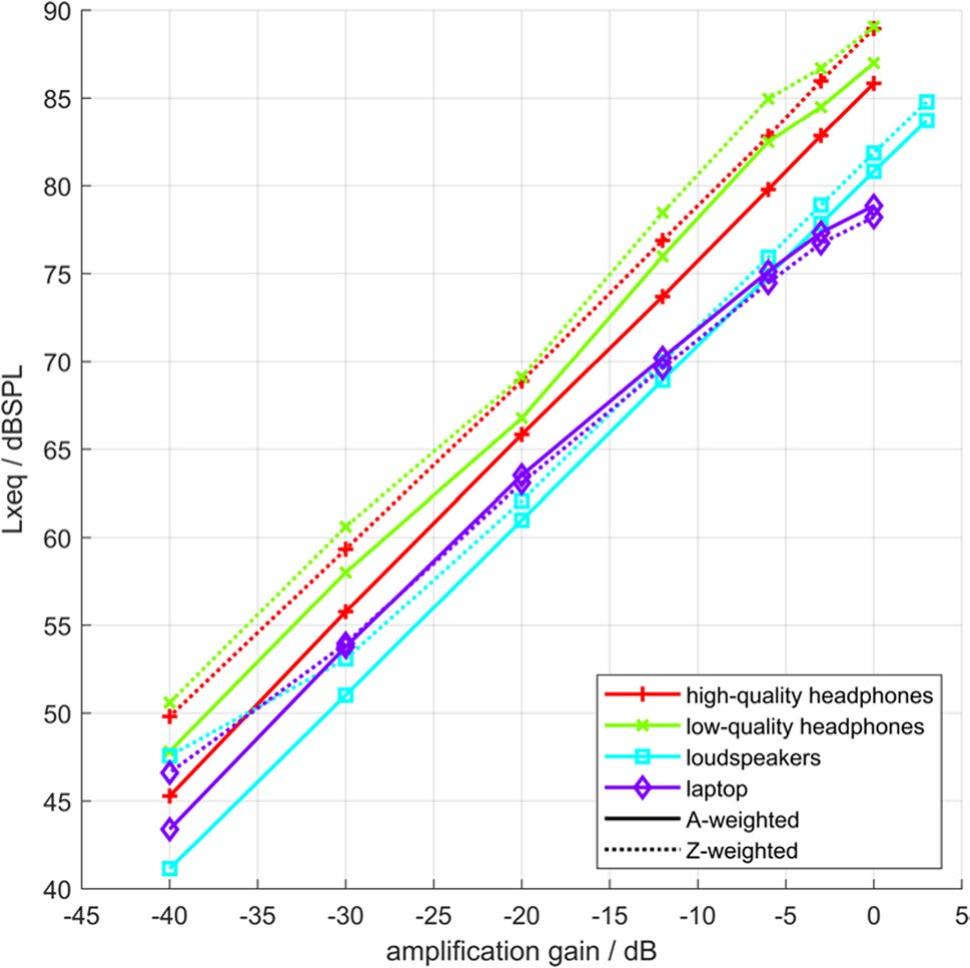
LAeq and LZeq as a function of gain for each playback device. *Note*. As the *y*-axis depicts both A- and Z-weighted levels (LAeq and LZeq), the level is denoted as Lxeq

Figure 4 shows the equivalent A- and Z-weighted sound pressure level LAeq and LZeq, respectively, as a function of the gain setting for the music stimulus. These values were based on the convolution of the individual impulse responses with the raw stimulus including crosstalk when appropriate. The differences between A- and Z-weighted levels of the individual devices mainly indicate low-frequency loss. Small differences between A- and Z-weighted levels indicate that only a small amount of low-frequency energy was reproduced, caused either by the stimulus itself or by the capabilities of the device. In case of the laptop, the A-weighted level was higher than the Z-weighted level, indicating dominant spectral energy between 1 and 6 kHz.

Nonlinear effects occurred at high amplification gains, especially for the laptop for gains from −12 dB to 0 dB and less so for the low-quality headphones for gains −6 dB to 0 dB. To determine the sound level as a function of gain adjusted by participants, we searched for the mathematical relation between dBFS values and dBSPL measurements. The HATS measurements for the right and left ears were averaged for each dBFS value. We aimed for the simplest regression equation that fitted the data with a coefficient of determination of *R*^2^ ≥ .99. For the loudspeaker and headphones, linear equations were sufficient. For the laptop, quadratic equations were used. We adjusted the *R*^2^ to take the complexity of the equation into account. As there were four transducer conditions and the two types of level (A- and Z-weighted), eight equations were used to estimate the sound levels set by the participants (see S5 in the Supplemental Materials for more details).

### Participants

The study was conducted in June and July, 2020. Participants were acquired through university mailing lists, advertising posters with a QR code, and social media posts. A total of 40 participants (mean age = 31.8 years, SD = 13.5, *n* = 15 male) took part in the study and gave written informed consent. Thirty-five participants reported normal hearing whereas five participants reported hearing loss (e.g., tinnitus, perception of noise). Each participant was paid €15 as reimbursement for participation. The study was performed in accordance with relevant institutional and national guidelines and regulations (Deutsche Gesellschaft für Psychologie, 2016; Hanover University of Music, Drama and Media, 2017) and with the principles outlined in the Declaration of Helsinki. Formal approval of the study by the ethics committee of the Hanover University of Music, Drama and Media was not mandatory, as the study adhered to all required regulations.

## Results

### Level adjustment

#### Comparison of heterogeneity

**Fig. 5:**
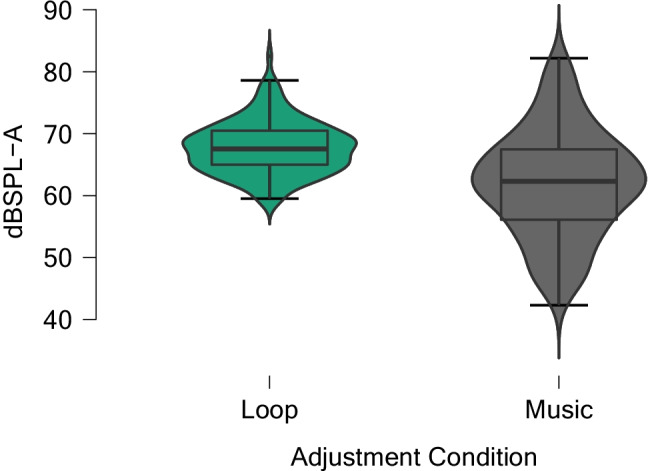
Violin plot of the adjusted sound pressure levels of participants with normal hearing (*n* = 35) over all playback devices (*n* = 4). *Note.* Each adjustment condition represents *n* = 140 measurement points (35 participants times four playback devices). dBSPL-A = A-weighted sound pressure level.

An important aim was the development of a procedure for reducing the heterogeneity in volume adjustments. For the 35 normal-hearing participants, we compared the two methods for level adjustment—Method 1 using the music stimulus and Method 2 using the loop method. For Method 1, participants adjusted to an average A-weighted SPL of 62.0 dB (median = 62.3 dB, min = 42.3 dB, max = 82.2 dB, SD = 8.7) as the preferred reproduction level. For Method 2, the selected average SPL of the respective music stimulus was 67.8 dB (median = 67.6 dB, min = 59.5, max = 82.6, SD = 4.3). The lower SD for the loop method indicates a decrease in heterogeneity. Both the SD and range were halved using the loop method. See Fig. 5 for a comparison of the two conditions. See Table 3 for descriptive statistics.

**Table 3:** Descriptive statistics for the adjustment conditions Loop and Music regarding the set dBSPL (A-weighted) by participants with normal hearing (*n* = 35)

	dBSPL-A	
	Loop	Music
Data points	140	140
Mean	67.8	62.0
Median	67.6	62.3
SD	4.3	8.7
Range	23.1	39.9
Minimum	59.5	42.3
Maximum	82.6	82.2
25th percentile	65.0	56.1
50th percentile	67.6	62.3
75th percentile	70.5	67.5

At the same time, the maximum level increased by a negligible amount (0.4 dB). To check whether the level adjustments followed a normal distribution, a Shapiro Wilk’s test was conducted for all conditions in both Method 1 and 2. At an alpha level of 5%, all tests revealed nonsignificant deviations (see S6 in the Supplemental Materials for details). Thus, the data were normally distributed in all transducer conditions and for both methods.

Levene’s tests were used to check whether the decrease in heterogeneity between Method 1 and Method 2 within the group of participants with no hearing loss (*n* = 35) was significant. The adjustments made by the music (Method 1) and loop (Method 2) stimuli were compared for every transducer condition. For all conditions, the test showed significant differences except for the laptop (MacBook Pro) condition (see Table 4). The variability ratio (ln VR) and the empirical coefficient of variation (v) [Nakagawa et al., 2015] were calculated to estimate the magnitude of heterogeneity reduction (see Table 5 and S6 in the Supplemental Materials for details).

**Table 4:** Results of Levene’s test regarding reduction of heterogeneity in level adjustments (A-weighted dBSPL) in participants with normal hearing (*n* = 35)

Transducer	*F*	*df*	*p*
Headphones, high-quality	10.637	1	.002
Headphones, low-quality	9.988	1	.002
Loudspeaker	14.080	1	<.001
Laptop	3.413	1	.069

**Table 5:** Variability ratio (ln VR) between music and loop method and empirical coefficient of variation (v) of the music (v_C_) and loop (v_E_) method regarding A-weighted dBSPL in participants with normal hearing (*n* = 35)

Transducer	ln VR	v_C_	v_E_
Headphones, high-quality	−0.711	0.124	0.058
Headphones, low-quality	−0.613	0.127	0.065
Loudspeaker	−0.702	0.134	0.054
Laptop	−0.433	0.104	0.064
Overall	−0.702	0.140	0.063

To assess the overall test–retest reliability (*r*_tt_) of Method 2 (loop) in all four playback conditions with JASP software (JASP Team, 2020), we conducted a Bayesian correlation analysis with uninformed priors (*N* = 160). The loop method was found to be highly reliable (*r*_tt_ = .899, 95% CI [.862, .924], BF_+0_ = 1.458e+55), as was the music condition (*r*_tt_ = .885, 95% CI [.843, .913], BF_+0_ = 1.013e+51). BF_+0_ indicates that the Bayes factor reports the evidence for the alternative hypothesis with a positive correlation over the null hypothesis.

#### Perceived loudness versus measured sound pressure level

Each time after adjusting the volume, participants evaluated the perceived loudness (categories: too soft, comfortable, too loud) of a reference stimulus (pop song). To analyze the perceived loudness after completing the loop method, we aggregated all loudness ratings (160 responses) related to the loop method for all participants (*N* = 40) and for all four devices.

**Fig. 6:**
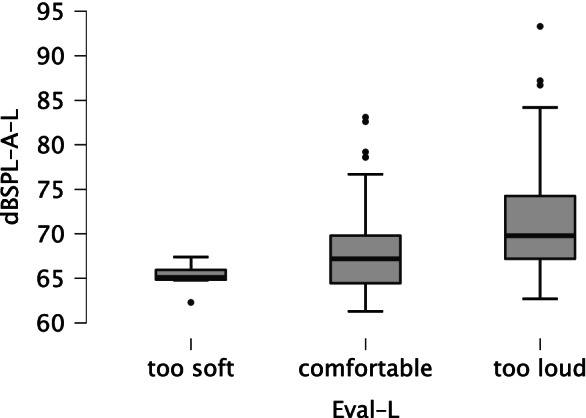
Boxplot of loudness ratings of a pop song after completing the loop method for level adjustment. *Note.* dBSPL-A-L = A-weighted sound pressure level after finishing the loop method. Eval-L = loudness evaluation of the pop song after finishing the loop method (*too soft*: *n* = 6, *comfortable*: *n* = 85, *too loud*: *n* = 69)

Figure 6 shows the distribution of A-weighted sound pressure levels for each loudness category. The sound pressure levels overlapped across categories. Therefore, we assume that evaluation differences were due more to individual preferences of loudness than to absolute level differences. Many participants evaluated the volume as being too loud, and only a few participants preferred volume levels above 85 dBSPL. The highest sound pressure level of 93.3 dB was selected by a participant with hearing loss. According to the criteria of the *National Institute for Occupational Safety and Health* (*NIOSH*), an exposure time of approximately 70 minutes would be permissible without causing damage (NIOSH, 1998) even for a level of 93.3 dB. In summary, the loop method did not seem to lead to harmful volume adjustments but may have conflicted with the loudness preferences of test subjects.

#### Determining participants’ adjustment accuracy

**Fig. 7:**
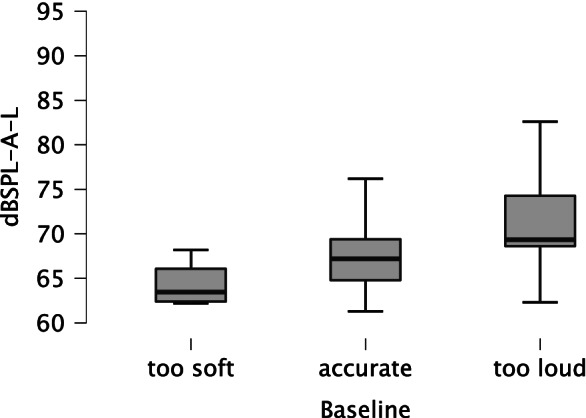
Boxplot of level distributions for the three loudness categories determined by the accuracy test (*too soft*: *n* = 6, *accurate*: *n* = 97, *too loud*: *n* = 37)

The analysis in the section *Comparison of heterogeneity* showed that the loop method can significantly reduce variability in level adjustments (see Table 4) and is highly reliable (*r*_tt_ = .899). Generally, after completing the loop method for the level adjustment, there still was variability in the level adjustments. By using another listening task, we tried to control for the remaining heterogeneity. The stimuli used consisted of noise events at three loudness levels. We compared the true sound levels set by the participants to the three loudness categories regarding the pop song (“too soft,” “accurate,” and “too loud”). Figure 7 shows the level distribution for all categories. A Spearman rank correlation for the calculation of test–retest reliability (*r*_tts_) for the participants with no hearing loss (*n* = 35) revealed a medium correlation (*r*_tts_ = .464, *p* < .001, 95% CI [.347, 1.00]). We concluded that the reliability of the baseline measurement (see section A.2 for details on baseline measurement) was low. Thus, with this method, it was not possible to acquire more information about participants' adjusted levels. Using the Spearman-Brown prophecy formula (Revelle & Condon, 2018) for attenuation correction, we found that the test length would have to be increased by five items (five times longer, *n*) to achieve an improved reliability (*r*_k_) of .812 (see Eq. 1).

1 $${r}_k=\frac{n\times {\overline{r}}_{ij}}{1+\left(n-1\right){\overline{r}}_{ij}}=\frac{5\times .464}{1+\left(5-1\right)\times .464}\approx .812$$

#### Manipulation check

To test whether the manipulation check could detect undesired volume changes, we used the categories determined by the accuracy task as a baseline measurement. Next, we examined whether a category deviating from the baseline was detected after a manipulated stimulus (softer and louder than right after completing the loop method) had been played back. We assumed that a softer playback causes the classification in a lower category than the baseline (too soft). In contrast, a louder playback will cause the classification in a higher category than the baseline (too loud). To investigate the precision of the manipulation check, we aggregated the answers for all four playback conditions. Participants with hearing loss were excluded. Due to the construction of the task, in some cases it is not possible to detect level changes. For example, very low playback levels did not allow for the detection of further decreases, and at very high playback levels, no further increase can be detected. Thus, these comparison pairs were excluded from the analysis. In *n* = 124 out of *N* = 237 cases (52.3%), the level manipulations were correctly detected. If no manipulation was applied, 68.6% of cases were correctly classified as “no manipulation detected.” The manipulation test built directly on the baseline measurement (Accuracy). As the reliability of the baseline measurement was limited, the reliability of the manipulation check must have been limited as well. Because of these weaknesses, we decided to exclude the control task for undesired level manipulations from the HALT procedure.

#### Low-frequency limits

The audibility of frequencies (20, 60, 100, 140 Hz) determined by HALT was compared with the measured reproduction levels of the respective frequencies for each playback device. As described previously, a quantification of low-frequency limits was proposed by determining the magnitude of falling below the spectral median. For this analysis, we designed a valuation criterion by taking the magnitudes below the median of the presented single-tone frequencies at 20, 60, 100, and 140 Hz and adding perception-based attenuations (−50.5 dB, −27.1 dB, −19.1 dB, −14.8 dB) from A-weighting (IEC, 2013). From the resulting data, we came up the criterion −40 dB relative to the spectral median as the listening threshold for detecting single tones (see Table 6). If the criterion value fell below −40 dB, the detection of single tones decreased. However, this assumption cannot be generalized due to the limited data basis of *N* = 40 participants. High test–retest reliability (*r*_tt_) was achieved in all four listening conditions across all four frequencies for the low-frequency test of the HALT procedure (*N* = 40 participants, *N* = 640 data points, *r*_tt_ = .821, 95% CI [.793, .844], BF_+0_ = 5.524e+153).

**Table 6:** Overview of low-frequency audibility determined by HALT and the respective measured valuation criterion below the spectral median

Transducer	20 Hz	60 Hz	100 Hz	140 Hz
Headphones, high-quality	0 (0%) −61.5 dB	40 (100%) −29.6 dB	40 (100%) −21.7 dB	40 (100%) −17.0 dB
Headphones, low-quality	0 (0%) −83.8 dB	26 (65%) −48.2 dB	39 (98%) −32.5 dB	40 (100%) −22.8 dB
Loudspeaker	0 (0%) −69.6 dB	40 (100%) −38.1 dB	40 (100%) −21.6 dB	40 (100%) −13.6 dB
Laptop	0 (0%) −65.5 dB	5 (13%) −48.4 dB	38 (95%) −41.2 dB	40 (100%) −28.6 dB

#### Mono/stereo playback

For all playback devices, we checked whether stereo playback could be detected by the HALT procedure. The responses of all participants (*N* = 40) were aggregated, resulting in 160 answers across all four playback conditions. Stereo playback was correctly identified 153 times (95.6%). The remaining responses indicated mono playback (*n* = 5), interchanged channels (*n* = 1), and miscounting (*n* = 1). A Bayesian Pearson correlation (Wagenmakers, Love, et al., 2018a; Wagenmakers, Marsman, et al., 2018b) was conducted to determine test–retest reliability (*r*_tt_) for detecting stereo playback, resulting in a high correlation (*r*_tt_ = .792, 95% CI [.722, .842], BF_+0_ = 5.704e+32). Therefore, we assumed that the reliability of the stereo detection fulfilled the standard benchmark of *r*_tt_ > .70 (Abell et al., 2009, p. 94).

## Discussion

In this project, we developed a headphone and loudspeaker test (HALT Part I) to remotely test characteristics of playback devices and listening conditions in internet experiments on auditory perception. In a laboratory study, listening tasks and procedure parts with insufficient reliability (*determining participants’ adjustment accuracy*, *manipulation check*) were identified and excluded from HALT Part I. We believe that due to their complexity, the excluded tasks were too difficult for participants. Additionally, the tasks were based on only one trial. By increasing the number of trials, it may be possible to improve the reliability. The final version of the HALT Part I procedure comprises three reliable main parts: level adjustment, mono/stereo playback, and determination of lower frequency limits. The suggested HALT Part I procedure takes approximately 3.5 minutes for completion.

Referring to the variability ratios and the empirical coefficients of variation, we argue that HALT reduces heterogeneity in reproduction level adjustments compared to conventional approaches by asking participants to adjust to a comfortable volume level. The test–retest analysis showed high reliability of level settings. As a negative side effect, some participants rated the loudness as too high after adjusting the volume according to the loop method. However, due to the relatively low level and expected exposure times, it was very unlikely that participation would result in hearing damage. But an uncomfortably high volume may affect the participants' experience of auditory stimuli. The responses of the participants regarding loudness were based on a reference song at −8.4 LUFS. When using HALT for level adjustments, researchers are not bound to a certain level for their stimuli. The participants’ responses and the LUFS value provide a reference point from which other levels can be set systematically. The standardized basic level of each test person remains unaffected. By lowering the overall volume of the stimuli (below −8.4 LUFS) we would expect an unknown number of subjects who evaluate the volume as being too low. Therefore, we suggest that the subjective experience of loudness should be noted when HALT is used. For practical applications, it is important to note that the playback level and the perceived loudness in our study were influenced by (a) the level of the adjustment stimulus in the loop method (−46 dBFS for soft noise events) and (b) the inherent loudness of the music stimulus (in our case, −8.4 LUFS). Users of HALT Part I who are aiming for a similar loudness in their study must adjust their stimuli to −8.4 LUFS. As we used a prototypical pop song for the loudness ratings, the digital level was close to 0 dBFS and, thus, the average loudness was expected to be evaluated as high. This leads to problems with classical music as this genre usually has a low average level (with a large dynamic range) but also contains passages that are close to 0 dBFS. In some cases, an adjustment of the stimulus is not possible because the digital level of 0 dBFS (clipping limit) would have to be exceeded to make the loudness of classical music match the loudness of pop music. To circumvent this problem, we have provided a second stimulus set for HALT, which has been standardized for stimuli with −20 LUFS. An online configurator will be provided which, among other things, can be used to select the volume standard (−8.4 LUFS or −20 LUFS).

The study was deliberately carried out in a non-optimized listening environment. Therefore, it cannot be ruled out that there will be response differences in acoustically optimized environments. For example, noise events might be audible at lower levels in such rooms, as fewer reflections and reverberations are expected that may affect the reproduction of the stimuli. It can be speculated that this may result in a lower volume level.

The procedure enables the detection of stereo playback and correct stereo channel assignment with high reliability.

The HALT procedure can estimate lower-frequency limits of playback devices. When laptops are used for playback, audible artifacts can occur so that counting tasks at various frequencies can be answered correctly without physical reproduction of the frequency under test by the laptop loudspeaker. Similar sound artifacts are expected for smartphones. Hence, the interpretation of the lower-frequency limit for these devices is limited. Therefore, smartphones must be further examined. More information about conclusive responses can be obtained by asking the participants about the manufacturer and model of the loudspeakers or headphones being used. Four playback devices were tested in our study. Targeted studies with laptops and, in particular, smartphones are necessary for researchers to better assess the reliability and generalizability of HALT.

Finally, we hope that the suggested procedures will contribute to improved data quality and efficiency in internet experiments on auditory perception. Data quality comparable to that of laboratory settings is a prerequisite for the future acceptance of internet listening experiments. In our upcoming paper “The Headphone and Loudspeaker Test – Part II” (Wycisk et al., 2021)*,* we address the question of how to apply screening methods to detect headphone and loudspeaker playback. Specifically, we introduce two new screening tests for the detection of headphone and loudspeaker playback. Building on the advantages of several different screening tests, we make suggestions as to how to apply strategic, mathematical, and statistical methods. The entire procedure of HALT Part I and Part II will be presented in the forthcoming paper HALT Part II. Additionally, we will provide an easy-to-use online configurator to set up the complete HALT (Parts I and II) for individual use.

## Supplementary Information

ESM 1 (PDF 33293 kb)
