# Supplementary material for: The Headphone and Loudspeaker Test – Part I: Suggestions for controlling characteristics of playback devices in internet experiments
Source: Behav Res Methods. 2022 May 17;55(3):1094–107. doi: 10.3758/s13428-022-01859-8 (PMC9113065; doi:10.3758/s13428-022-01859-8)
Supplement: Supplementary file 1 — (PDF 33293 kb) [file 13428_2022_1859_MOESM1_ESM.pdf]

Supplemental Materials

The Headphone and Loudspeaker Test – Part I: Suggestions to Control Characteristics of Playback Devices in Internet Experiments

Yves Wycisk, Reinhard Kopiez, Jakob Bergner, Kilian Sander, Stephan Preihs, Jürgen Peissig, Friedrich Platz

Content: **Page**

S1. Room Acoustic Measurements of the Hanover Music Lab	2
S2. Photos of the experimental Setup	3
S3. Sequence Plans for HALT – Part I conducted in the Laboratory	5
S4. Flowchart of the Signal Chain	6
S5. Transforming dBFS to dBSPL by Curve Fitting	7
S6. Impact of the Adjustment Procedure on the Dispersion of dBSPL (A)	8

S1. Room Acoustic Measurements of the Hanover Music Lab

(a) Dimensions

Width: 4.26 m, Length: 5.38 m, Height: 2.69 m

(b) Background Noise

Duration of measurement: 1 minute.

L_{Aeq} = 30.1 dB
L_{Ceq} = 38.1 dB
L_{Zeq} = 42.1 dB
L_{AFmax} = 42.9 dB
L_{ZFmax} = 51.9 dB

(c) Reverberation Time (RT60)

Hardware Configuration

Device Info:	NTi Audio XL2
Mic Type:	NTi Audio M4260
Mic Sensitivity:	33.4 mV/Pa

Measurement Setup

Profile:	Full mode
Resolution:	1/3 Octave
Range:	40 - 140 dB

RT60 Average Results for Three Measurements. Overall Average Reverberation Time: 0.57 seconds

Band [Hz]	RT60 (T30) [s]	MeasUnct [%]
50	0.27	38.22
63	0.78	20.06
80	0.37	25.72
100	0.51	19.71
125	0.74	14.64
160	0.63	14.03
200	0.75	11.50
250	0.79	10.04
315	0.76	9.08
400	0.70	8.44
500	0.62	8.00
630	0.58	7.35
800	0.53	6.82
1000	0.53	6.14
1250	0.57	5.27
1600	0.55	4.75
2000	0.56	4.22
2500	0.55	3.81
3150	0.53	3.45
4000	0.52	3.09
5000	0.50	2.82
6300	0.46	2.61
8000	0.43	2.41
10000	0.35	2.37

S2. Photos of the measurement and experimental Setup

A: overview, floor marks

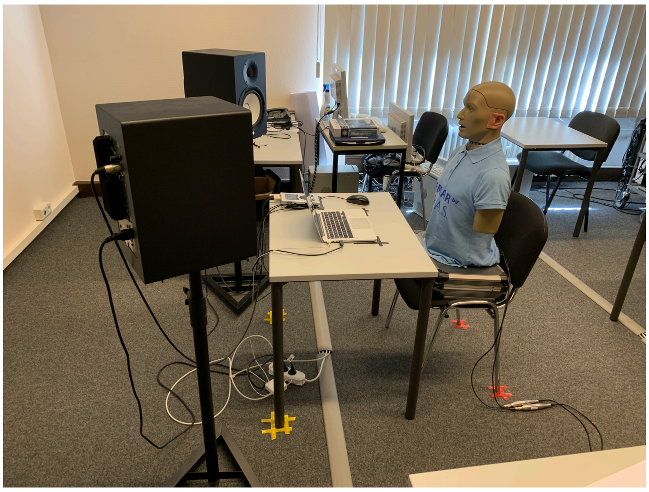

B: overview, headphone measurement

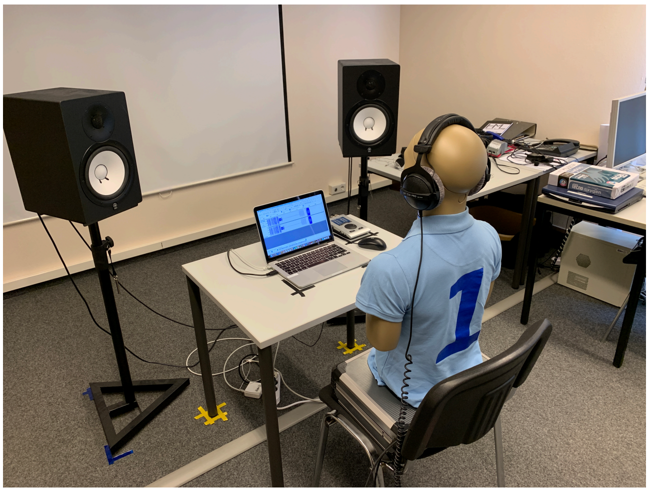

C: close up, table, device setup/marks

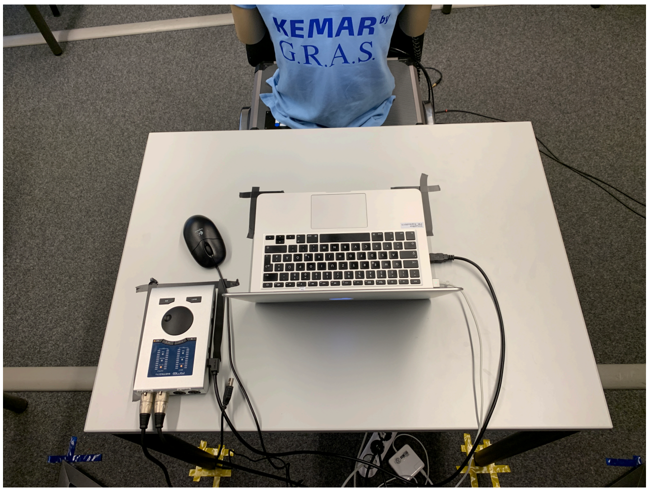

D: close up, device setup

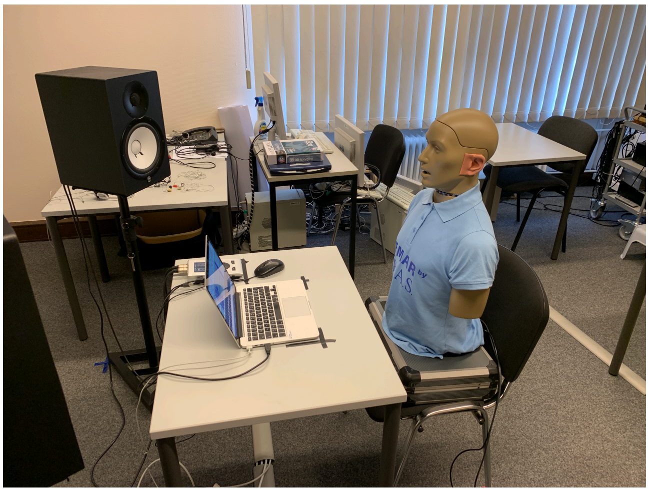

E: overview, room

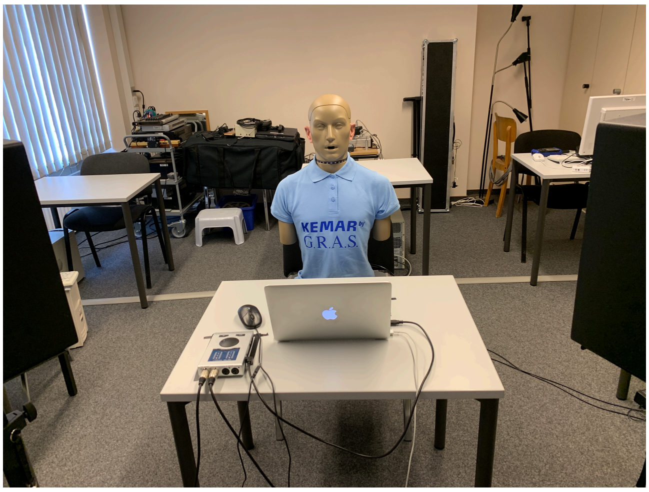

F: close up, headphone measurement

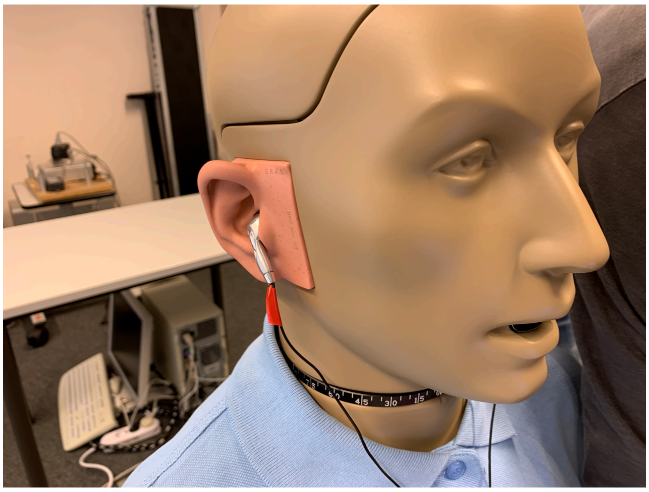

G: close up, device setup/marks

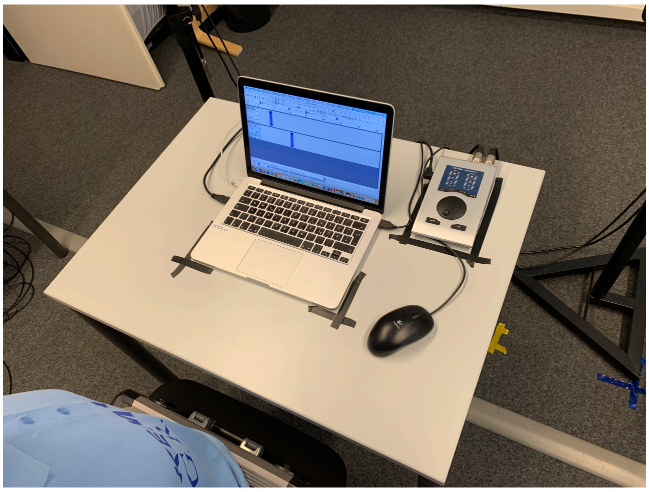

H: close up, HATS, side/rear

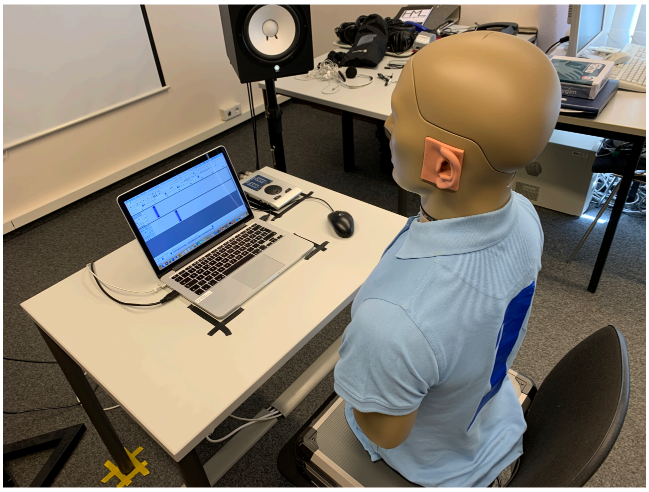

I: close up, HATS, side/front

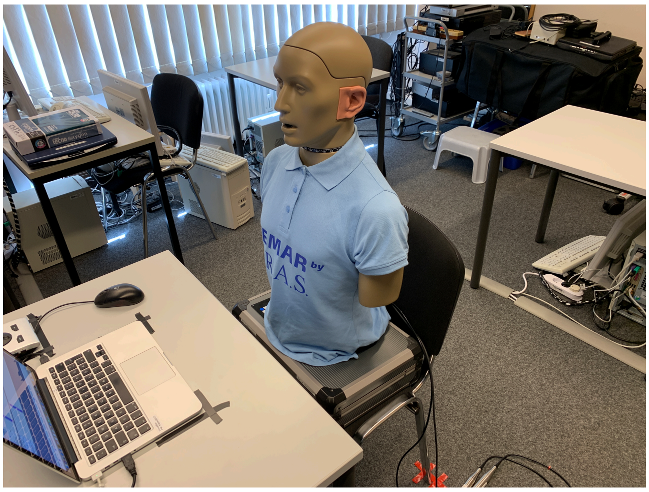

J: close up, device setup/marks

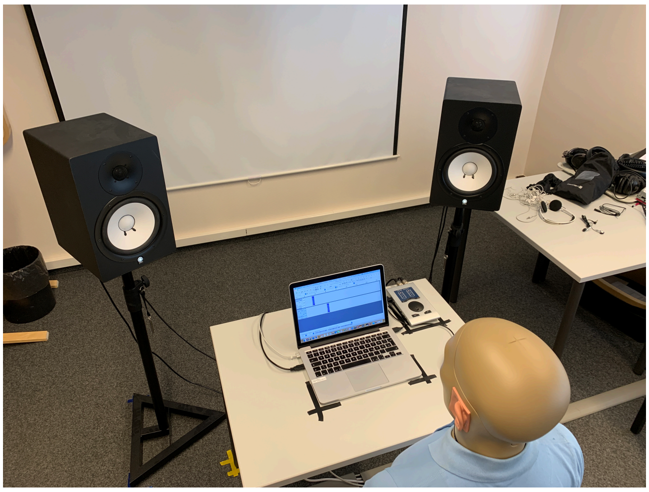

S3. Sequence Plans for HALT Part I conducted in the Laboratory

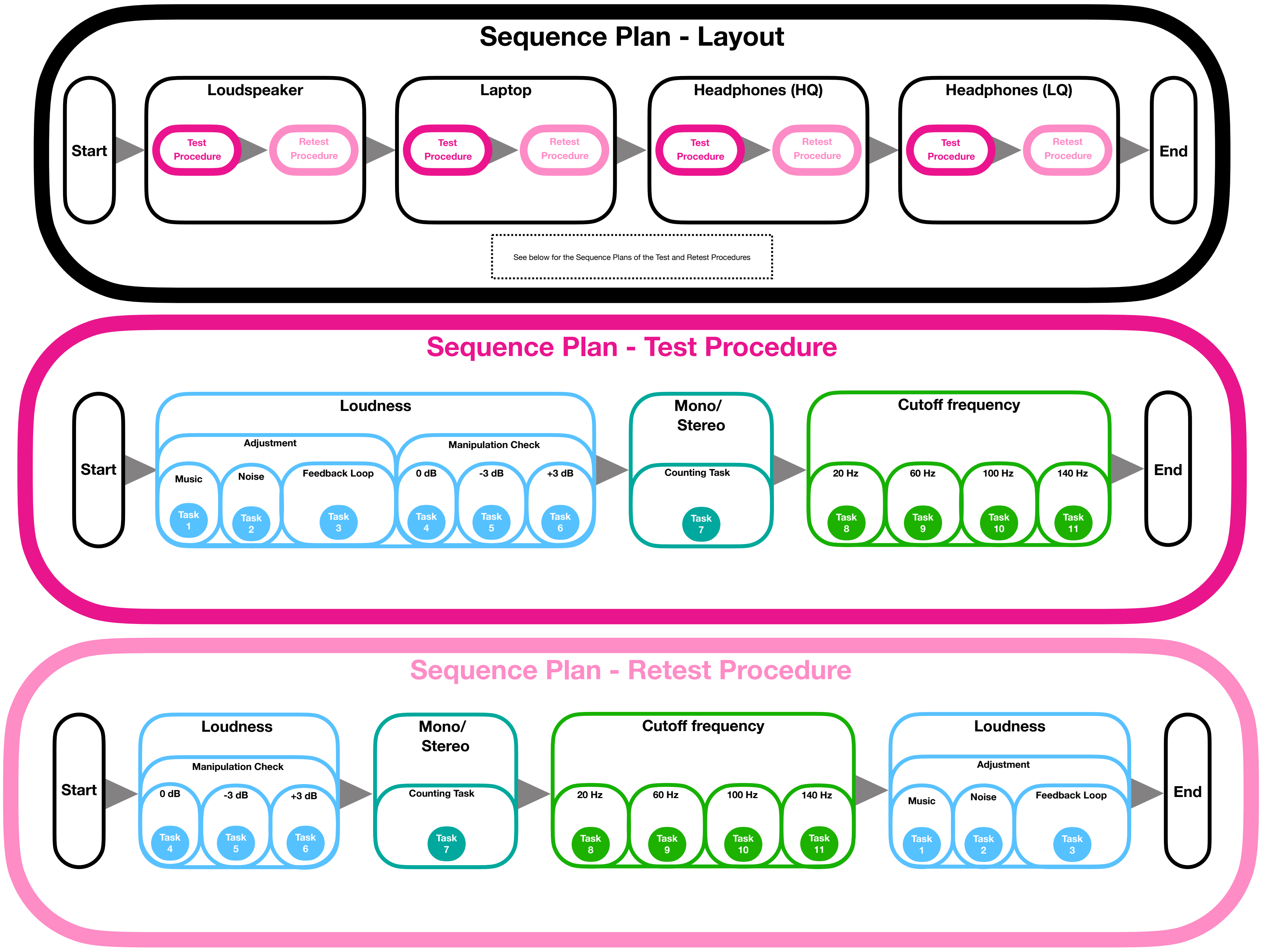

S4. Flow Chart of the Signal Chain

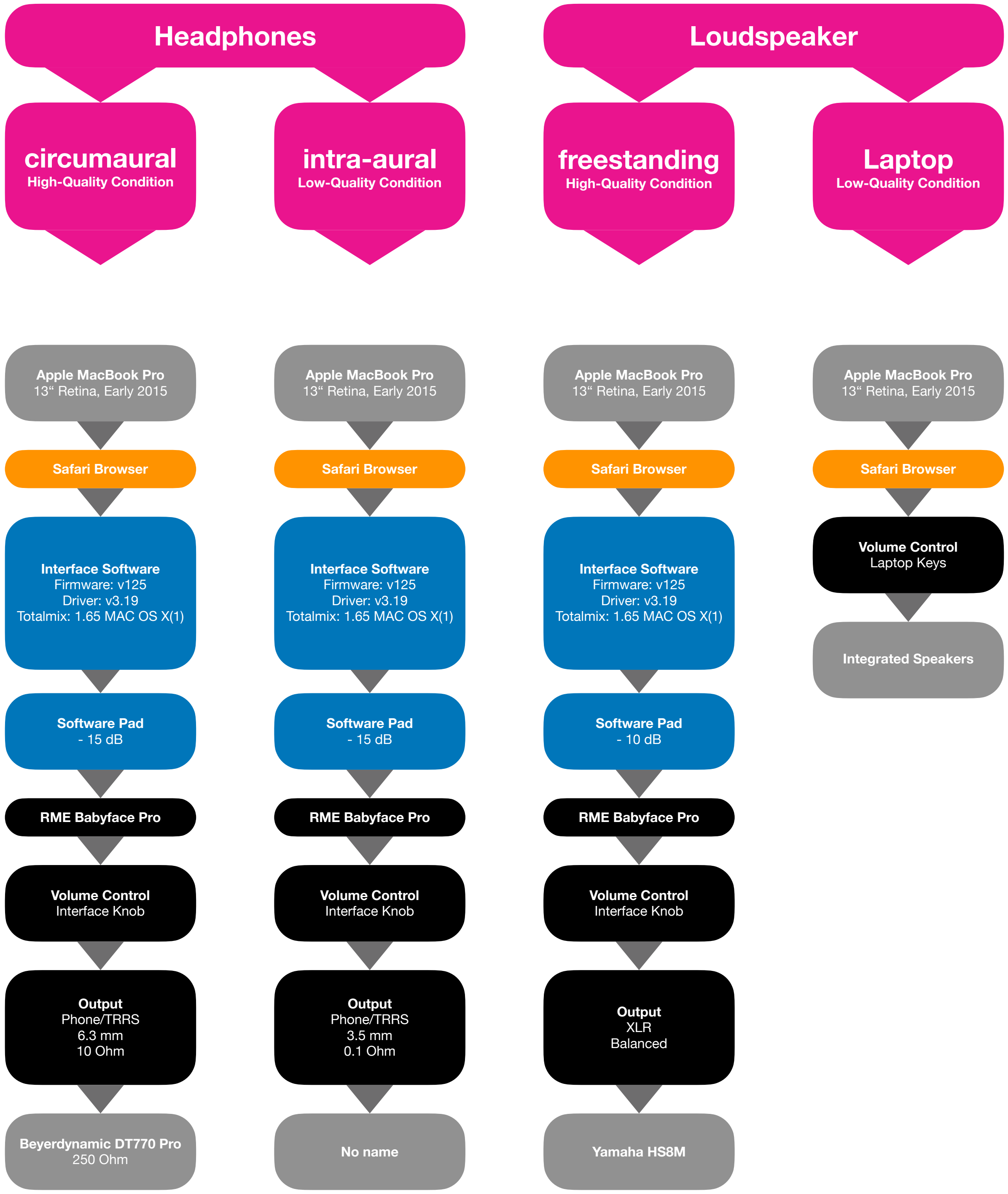

S5. Transforming dBFS to dBSPL by Curve Fitting

Identified Equations for each Playback Device

Headphones, high-quality:

dBSPL(A), $y = 0.9994x + 86.233$

dBSPL(Z), $y = 0.9898x + 89.153$

Headphones, low-quality:

dBSPL(A), $y = 1.0173x + 87.203$

dBSPL(Z), $y = 1.01x + 89.387$

Loudspeaker:

dBSPL(A), $y = 0.9983x + 81.202$

dBSPL(Z), $y = 0.9432x + 81.834$

Laptop:

dBSPL(A), $y = -0.0054x^2 + 0.6821x + 79.456$

dBSPL(Z), $y = -0.0033x^2 + 0.7253x + 78.928$

Exact R^2 values calculated both for A-/Z-weighted dBSPL.

Transducer	Brand/Modell	R^2(A)	R^2_{adj}(A)	R^2(Z)	R^2_{adj}(Z)
Headphones, high-quality	Beyerdynamic, DT 770 Pro, 250 Ohm	.9999	.9999	.9999	.9999
Headphones, low-quality	No name	.9972	.9966	.9959	.9951
Loudspeaker	Yamaha HS8M	.9995	.9994	.9984	.9981
Laptop	Apple MacBook Pro, retina, 13", early 2015	.9998	.9997	.9994	.9991

S6. Impact of the Adjustment Procedure on the Dispersion of dBSPL (A)

Preliminary Considerations for Normal-Hearing Participants (*N* = 35)

Shapiro-Wilk's Test

Test of Normality (Shapiro-Wilk)

		W	p
Headphones, high-quality, dBSPL (A)	Loop	0.970	.435
	Music	0.987	.946
Headphones, low-quality, dBSPL (A)	Loop	0.939	.052
	Music	0.970	.433
Loudspeakers, dBSPL (A)	Loop	0.944	.073
	Music	0.976	.613
Laptop, dBSPL (A)	Loop	0.940	.055
	Music	0.967	.374

Note. Significant results suggest a deviation from normal distribution. At an alpha level of .05, it is assumed that the data are normally distributed.

Levene's Test

Test of Equality of Variances (Levene's Test)

	F	df	p
Headphones, high-quality, dBSPL (A)	10.637	1	.002
Headphones, low-quality, dBSPL (A)	9.988	1	.002
Loudspeakers, dBSPL (A)	14.080	1	< .001
Laptop, dBSPL (A)	3.413	1	.069

Note. Significant results suggest that the loop condition was able to reduce the deviation in adjusted sound pressure levels compared to the music condition. At an alpha level of .05, no significant difference regarding the laptop condition is assumed.

Magnitude of Dispersion Reduction for Normal-Hearing Participants (*N* = 35) Applying the Variability Ratio

$\ln\sigma \triangleq$ natural logarithm of the population standard deviation

$\mathrm{s} \triangleq$ standard deviation

$\ln\hat{\sigma} = \ln s + \frac{1}{2(n-1)}$ $\triangleq$ estimation of $\ln\sigma$

$s^2_{\ln\hat{\sigma}} = \frac{1}{2(n-1)}$ $\triangleq$ sampling variance

$n_E = 35 \triangleq$ sample size of the experimental group (140 for overall calculation)

$n_C = 35 \triangleq$ sample size of the control group (140 for overall calculation)

$\ln \mathrm{VR} = \ln\left(\frac{\mathrm{s_E}}{\mathrm{s_C}}\right) + \frac{1}{2(n_E-1)} - \frac{1}{2(n_C-1)}$ $\triangleq$ variability ratio

Interpretation: A lower value of the variability ratio indicates a better reduction in dispersion. For example, when high-quality headphones were used, the dispersion was smaller compared to the laptop condition.

Headphones, high-quality, dBSPL (A):

$$\ln \mathrm{VR}_{Hp-A} = \ln\left(\frac{\mathrm{s_E}}{\mathrm{s_C}}\right) + \frac{1}{2(n_E-1)} - \frac{1}{2(n_C-1)} = \ln\left(\frac{3.963}{8.070}\right) + \frac{1}{2(35-1)} - \frac{1}{2(35-1)} = -0.711$$

Headphones, low-quality, dBSPL (A):

$$\ln \mathrm{VR}_{Hp-B} = \ln\left(\frac{\mathrm{s_E}}{\mathrm{s_C}}\right) + \frac{1}{2(n_E-1)} - \frac{1}{2(n_C-1)} = \ln\left(\frac{4.513}{8.327}\right) + \frac{1}{2(35-1)} - \frac{1}{2(35-1)} = -0.613$$

Loudspeakers, dBSPL (A):

$$\ln \mathrm{VR}_{Ls} = \ln\left(\frac{\mathrm{s_E}}{\mathrm{s_C}}\right) + \frac{1}{2(n_E-1)} - \frac{1}{2(n_C-1)} = \ln\left(\frac{3.653}{7.369}\right) + \frac{1}{2(35-1)} - \frac{1}{2(35-1)} = -0.702$$

Laptop, dBSPL (A):

$$\ln \mathrm{VR}_{Lt} = \ln\left(\frac{\mathrm{s_E}}{\mathrm{s_C}}\right) + \frac{1}{2(n_E-1)} - \frac{1}{2(n_C-1)} = \ln\left(\frac{4.178}{6.442}\right) + \frac{1}{2(35-1)} - \frac{1}{2(35-1)} = -0.433$$

Overall, dBSPL (A):

$$\ln \mathrm{VR}_{all} = \ln\left(\frac{\mathrm{s_E}}{\mathrm{s_C}}\right) + \frac{1}{2(n_E-1)} - \frac{1}{2(n_C-1)} = \ln\left(\frac{4.287}{8.648}\right) + \frac{1}{2(140-1)} - \frac{1}{2(140-1)} = -0.702$$

Magnitude of Dispersion Reduction for Normal-Hearing Participants (*N* = 35) Applying the Empirical Coefficient of Variation

$\mathrm{v} = \frac{s}{\bar{x}} \triangleq$ coefficient of variation

$\mathrm{v}_E \triangleq$ coefficient of variation in loop condition

$\mathrm{v}_C \triangleq$ coefficient of variation in music condition

$\bar{\mathrm{x}} \triangleq$ mean

$\mathrm{s} \triangleq$ standard deviation

Interpretation: A lower value of the empirical coefficient of variation indicates a better reduction in dispersion. For example, when high-quality headphones were used, the dispersion was smaller compared to that under the laptop condition.

Headphones, high quality, dBSPL (A):

$$\mathrm{v}_E = \frac{s_E}{\bar{x}_E} = \frac{3.963}{68.7} = 0.0576$$

$$\mathrm{v}_C = \frac{s_C}{\overline{x_C}} = \frac{8.070}{65.3} = 0.1236$$

Headphones, low quality, dBSPL (A):

$$\mathrm{v}_E = \frac{s_E}{\bar{x}_E} = \frac{4.513}{69.194} = 0.0652$$

$$\mathrm{v}_C = \frac{s_C}{\overline{x_C}} = \frac{8.327}{65.834} = 0.1265$$

Loudspeaker, dBSPL (A):

$$\mathrm{v}_E = \frac{s_E}{\bar{x}_E} = \frac{3.653}{67.686} = 0.0540$$

$$\mathrm{v}_C = \frac{s_C}{\overline{x_C}} = \frac{7.369}{55.094} = 0.1338$$

Laptop, dBSPL (A):

$$\mathrm{v}_E = \frac{s_E}{\bar{x}_E} = \frac{4.178}{65.509} = 0.0638$$

$$\mathrm{v}_C = \frac{s_C}{\overline{x_C}} = \frac{6.442}{61.729} = 0.1044$$

Overall, dBSPL (A):

$$\mathrm{v}_E = \frac{s_E}{\bar{x}_E} = \frac{4.287}{67.772} = 0.0633$$

$$\mathrm{v}_C = \frac{s_C}{\overline{x_C}} = \frac{8.648}{61.989} = 0.1395$$
